# Using PlayWorld to Promote Narrative Development: Evidence from a Double-blind Control Experiment

**DOI:** 10.11621/pir.2025.0309

**Published:** 2025-09-30

**Authors:** Nikolay N. Veresov, Aleksander N. Veraksa, Valeriya A. Plotnikova

**Affiliations:** a Monash University, Melbourne, Australia; b Lomonosov Moscow State University, Russia

**Keywords:** pretend play, PlayWorld, coherent speech, narrative competence, cultural-historical approach

## Abstract

**Background:**

Recent data indicate an increase in speech difficulties and a decline in narrative competence among today’s preschool children. Therefore, identifying effective methods to support the development of narrative competence is a pressing and relevant challenge.

**Objective:**

The aim of this study was to evaluate the efficacy of using PlayWorld^1^ interventions in fostering narrative competence in preschool children.

**Design:**

The study involved 90 children aged 5–6 years and compared: (1) Play-World— a form of joint child-adult pretend play based on a fairy tale plot, (2) free pretend play, and (3) a control group. The research employed a randomised controlled trial design. Children’s narratives were assessed using the “MAIN: Multilingual Assessment Instrument for Narratives”, focusing on word count, speech rate, and both macrostructure (semantic level) and microstructure (lexical-grammatical level) of narrative production.

**Results:**

The results revealed that children receiving PlayWorld interventions significantly improved their macro- and microstructure of narratives, whereas in free pretend play children improved only the macrostructure of narratives. Children in the control group showed significant decline of scores for the macrostructure of narratives.

**Conclusion:**

The findings revealed that PlayWorld interventios are an effective approach for developing narrative competence. The use of cultural texts and adult involvement in pretend play are important complementary factors that enhance the developmental impact of pretend play. The findings contribute to a more precise understanding of how pretend play supports narrative development and may have both theoretical and practical implications for future research and educational practice.

## Introduction

In an age of reduced book reading and increased screen exposure, young children often receive less structured language input. This shiftmay hinder the development of narrative competence, particularly during the critical period of narrative skill formation around the ages of 5 to 6. For example, a large Russian study found that preschoolers’ overall screen time was unrelated to their vocabulary size but negatively related to their ability to construct coherent spoken stories (Oschepkova & Shatskaya, 2023; Oschepkova et al., 2025). According to recent data from the World Health Organisation and Matsuo et al. (2024), the proportion of children experiencing speech and language difficulties is steadily rising: approximately 58 % of preschool- and early-primary-aged children now present with such challenges, and the absolute number of children with clinically diagnosed speech disorders has increased more than six-fold over the past two decades. At the same time, numerous reports have documented a long-term drop in children’s leisure reading worldwide, prompting educational policies to reemphasise early reading and storytelling (Klopotova & Smirnova, 2024; Kortava, 2024). These trends, reflecting the changing conditions of contemporary childhood, have intensified interest in evidence-based approaches for supporting children’s narrative competence.

Shared book reading with an adult and “dialogic” reading are most well-documented strategies. Research show that frequent joint reading provides powerful language input and rich storytelling models, improving literal understanding of stories, as well as breadth and depth of vocabulary (Grolig et al., 2019; Kamalova, 2024; O’Farrelly et al., 2018). Even a single story read aloud can introduce new vocabulary and complex sentence structures that children would not hear in everyday life (Rivera Valdez & López Cortés, 2024; Salley et al., 2022). Many successful interventions combine reading and active storytelling practice. A recent systematic review (meta-analysing 26 studies) found that narrative-focused programs (often with manualised curricula and use of authentic children’s literature) produced significant gains in children’s narrative skills (Pico et al., 2021). However, these approaches present several important limitations. Shared book reading with an adult and “dialogic” reading offer limited opportunities for children to invent or adapt content, often resulting in reduced spontaneity and diminished creative engagement. Moreover, preschool-aged children frequently perceive such formulaic tasks as uninteresting or repetitive. In practice, teacher-centered reading sessions or drill-based language exercises may fail to sustain children’s attention and motivation.

In contrast, research has shown that embedding storytelling within playful, child-centered contexts significantly enhances engagement. For example, Chlapana and Koniou (2025) conducted experiments with 32 children aged 4-6 years who participated in classroom group activities for one month, namely interactive reading and reading with dramatisation. It was found that the use of props and dramatisation in storytelling not only increased children’s enjoyment but also boosted their motivation to participate actively in the learning process. Moreover, in a study conducted by Oshchepkova and colleagues (2023), 220 children (M = 60.84 months, SD = 4.14 months) participated in group activities, including pretend play, digital games, games with rules, reading and drawing stories. The sessions were conducted twice a week over a period of seven weeks. Results showed that pretend play benefits narrative competences of preschoolers more than reading and digital games. In this light, pretend play can serve as a compelling alternative to traditional shared reading and structured narrative exercises, offering a more autonomous and intrinsically motivating pathway for the development of narrative competence.

### Pretend play and narrative development: cultural-historical approach

According to the cultural-historical approach, pretend play is the leading activity for children aged 3 to 7 years and has the most significant impact on the mental development at this period (Bodrova et al., 2019; Elkonin, 2005). Pretend play is a culturally determined type of child’s activity, where they reenact various areas of real life in conditioned situations, that is, they master social roles and communication skills. The great advantage of play is that, compared to other means of improving speech and language, it takes place not as series of lessons or drills in isolated skills but as a social necessity, everyday communication activity; brings pleasure, but also benefits development (Gavrilova et al., 2025; Vygotsky, 2017).

Three key aspects of pretend play determine its potential for narrative development: (1) accepting roles (Elkonin, 2005; Veraksa et al., 2021), (2) imaginary situation (Veraksa, 2022; Vygotsky, 2017), and (3) symbolic nature of play (Veraksa, 2011; Vygotsky, 2017). Accepting roles and imaginary situation requires children to engage in two forms of communication: (1) communication about the play— children negotiate the roles, plot, how to organise the game etc. (2) the communication held within the play, in which the children relate to one another in the roles they agreed to perform (Bluiett, 2009; Fein, 1979). In pretend play children construct narrative structure, for example, they sequence events, establish causal relationships, and express intentions. Role-based dialogue in pretend play encourages children to adopt multiple perspectives and engage in extended, coherent conversations. Symbolic transformation— the ability to use objects and actions to represent other things— has also been linked to the development of more flexible and abstract language use (Lillard et al., 2013; Vygotsky, 2011). Thus, joint pretend play serves as an effective scaffold for the development and acquisition of narrative competence in preschool-aged children.

However, these mechanisms are more fully activated in the context of mature forms of pretend play. Rich and developed pretend play demands a lot from a child: to have an idea of the diversity of the surrounding reality, be familiar with a wide range of characters and be able to create and keep in mind an imaginary situation, come up with and develop a plot, accept and follow a chosen character, organise the playing space and select appropriate attributes, use substitute objects, and cooperate with peers (Elkonin, 2005; Veraksa, 2022). An adult as a carrier of cultural experience and knowledge can enrich children’s play, give examples of actions, roles, and plots. Therefore, the participation of an adult in children’s pretend play can affect its course and richness and, therefore, to some extent, influence the speech development in children (Veresov et al., 2021).

To conclude, pretend play contains developmental mechanisms that effectively support the growth of narrative competence in preschool children. However, an important question remains: can the combination of pretend play and shared reading enhance this developmental impact even further?

### Main features of PlayWorld

The PlayWorld framework represents an educational practice that integrates joint adult–child pretend play with shared reading of a cultural text as its foundation (Fleer, 2022; Hakkarainen & Bredikyte, 2020). The methodological foundation of this approach is grounded in the principles of cultural-historical theory, which emphasises the social and symbolic nature of child development. Central to this framework is the creation of a shared fictional world— a playworld— where both children and educators actively participate, blurring the traditional boundaries between teacher and student roles (Hakkarainen et al., 2013; Veraksa et al., 2023).

Adult partnership with children in early childhood education presents a significant challenge. Educators often perceive their role in children play as an advisor, supervisor, observer or organiser, rather than play partner. PlayWorld require both active and genuine adult participation in children play as a play partner and as a play guide. Genuine participation in children role play allows to keep children in an imaginary situation for longer time, give examples of play actions, share cultural experience, and promote greater emotional involvement of children. Children and educators co-create and participate in shared fictional contexts, allowing exploration of various roles and plots. Being a play guide implies providing structure and scaffolding to support learning. Capturing both perspectives helps to enrich play plot and role actions, enhance social interaction.

In PlayWorld, storytelling serves as an element, providing a framework for the play scenarios. Before the play starts, an adult and children read a story or a fairytale, which reflects the children’s cultural backgrounds. A story or a fairytale serves as a starting point for the pretend play and a resource for its development. However, a flexible interpretation of the storyline is allowed and even encouraged. Cultural texts, such as folktales or fairytales, hold significant developmental value as they present emotionally relevant conflicts and challenges that align with a child’s psychological development (Baumer et al., 2005). These stories often depict common experiences, including separation, jealousy toward siblings, anger toward parents. Additionally, the exaggerated characterisation of protagonists and antagonists (*e.g*., good versus evil, bravery versus cowardice) enhances the emotional intensity of these texts, reinforcing moral distinctions and fostering communication, discussions (Melik-Pashayev, 2025).

Despite a substantial body of theoretical literature and qualitative research supporting the potential of the Playworld framework, there is a notable lack of empirical data on its effectiveness for fostering narrative development in children. To date, only one quasi-experimental study has directly examined this question. Baumer et al. (2005) conducted a 14-week intervention involving 20 children (M = 5.5 years), where the experimental group participated in PlayWorld activities. The control group engaged in more traditional literacy practices typical of the classroom: teacher read-alouds, class discussions, partner and independent reading of level-appropriate picture books, and individual writing with accompanying illustrations. Children in the PlayWorld group showed significant gains in narrative length, coherence, and comprehension compared to the control group. However, the extent to which the PlayWorld framework supports the development of narrative competence remains underexplored. In particular, further research is needed to clarify the specific contribution of integrated elements such as shared reading, adult participation, and pretend dramatisation of cultural texts in shaping children’s narrative competence.

### Current study

The aim of this study was to evaluate the efficacy of PlayWorld interventions in fostering narrative competence in preschool children. Specifically, the study compares the effectiveness of the PlayWorld framework with free pretend play. To address the research goal, a double-blind randomised control experimental trial was organised.

In this study, narrative competence is understood as comprising both the semantic and lexico-grammatical levels of speech production. The semantic level of a narrative, or macrostructure, encompasses pragmatic aspects of speech such as coherence, integrity, completeness, adequacy, conclusiveness, and structural organisation. The lexical-grammatical level, or microstructure, refers to the appropriate use of linguistic means to convey meaning. The microstructure of a narrative includes lexical diversity, correct word usage, proper grammatical sentence construction, syntactic variety, and agreement of word forms, among other linguistic features.

According to the cultural-historical theory (Vygotsky, 1978; Verenikina, 2003), as to the idea of cooperation and communication with an adult as a condition for the child development, three specific hypotheses were formulated:

1. The developmental effect of PlayWorld framework on the length of narratives will be greater than in free play and control group.
2. The developmental effect of PlayWorld framework on the macrostructure of narratives will be greater than in free play and control group.
3. The developmental effect of PlayWorld framework on the microstructure of narratives will be greater than in free play and control group.

## Methods

### Participants

The study was carried out in 2024. The overall sample comprised 90 children aged 5 to 6 years (M= 70.3 months, SD = 3.6 months, Range = 27 months) attending federally funded kindergartens in Moscow, Russia, at the time of the pre-test. A total of 23 children were excluded from the analysis due to missing post-test data (n = 13) or attending fewer than half of the sessions (n = 10). A significant number of children did not participate in the post-test as there was a measles epidemic in Moscow kindergartens in December 2024 .The number of children in the final sample under the analysis was 67 (M = 69.8 months, SD = 4.1 months, Range = 18 months), of whom 33 (49.2%) were male and 34 female (50.8%).

The parents of each participant gave their written consent for their child to participate in the study and for video recording. The sample size was determined by the number of kindergartens and teachers who allowed the intervention. Not all children from the classes participated in the intervention, some just followed the usual curriculum. This allowed the formation of groups balanced in sex and age. The study was approved by the Ethics Committee of the Faculty of Psychology of Lomonosov Moscow State University.

### Procedure

The study was conducted using a randomised double-blinded experimental design with repeated measures. It had several stages. First, children’s level of narrative development was individually assessed using the “MAIN: Multilingual Assessment Instrument for Narratives” (pre-test). The assessment was conducted individually in a quiet, familiar environment for the child. It was administered by specially trained psychologists who were not involved in the intervention sessions.

The transcription of the audio recordings of the narratives and their evaluation were carried out by one expert who had received training from the MAIN developers. The expert was not aware of the purpose of the study and did not participate in either the assessment or the experiment. Then children were divided into 3 groups: PlayWorld group, Free Play group, Control group. Groups did no differ in the initial level of narrative development (ANOVA, F(2,54) = .714, p = .494 for the word count in narratives; F(2, 54) = .58, p = .563 for the macrostructure of narratives; F(2, 54) = .972, p = .31 for the microstructure of narratives).

An 11-week long intervention began one week after the pre-test. The sessions were held twice a week and lasted 20–30 minutes each. A total of 22 sessions were conducted in both the PlayWorld and Free Play groups. Each experimental condition included three subgroups of 10 children (three subgroups in the PlayWorld group and three subgroups in the Free Play group). The sessions were conducted by three specially trained experimenters with backgrounds in psychology and education. To control for experimenter effects, each experimenter led sessions in one PlayWorld subgroup and one Free Play subgroup. The sessions were conducted in familiar preschool settings, such as classrooms used for extra-curriculum activities. The environment was familiar and comfortable for the children. Sessions 4–6, 9, and 18–20 were video-recorded using a smartphone. The assessment of the video recordings was conducted with the primary aim of providing feedback to the participating experimenters and supporting them in overcoming difficulties. In particular, attention was given to how the adult could integrate more naturally into children’s pretend play as an equal participant, at which points the adult tended to step out of role, and how to enhance engagement. Experimenters documented children’s attendance at each session. If a child did not wish to participate in the play activity, they were offered the option to return to the classroom. Attendance records by subgroups, ensuring anonymity, are presented in *Table 1*.

**Table 1:** Attendance records by subgroups

Session N°/Subgroup	1	2	3	4	5	6	7	8	9	10	11	12	13	14	15	16	17	18	19	20	21	22
		Number of children present at the session (max. 10)																					
PlayWorld Subgroup 1	6	4	5	6	7	6	5	5	4	7	6	8	5	6	6	4	7	6	6	8	5	6
PlayWorld Subgroup 2	10	9	5	8	9	9	10	8	10	8	9	9	9	5	7	5	5	9	10	8	8	9
PlayWorld Subgroup 3	9	10	9	10	7	9	9	8	8	7	9	5	7	7	10	9	6	4	5	4	9	9
Free Subgroup Play 1	6	8	10	10	7	10	9	7	6	9	7	6	9	10	10	8	6	8	9	10	6	7
Free Subgroup Play 2	7	6	8	5	7	3	7	4	7	5	9	6	7	5	7	6	9	6	8	7	7	7
Free Subgroup Play 3	10	7	9	6	6	10	10	6	10	10	10	4	3	8	9	10	10	7	9	6	9	8

The post-test, similar to the baseline diagnostics (pre-test), was administered within 10 days following the completion of the intervention.

### Narrative assessment

The assessment of the speech development in preschoolers was carried out using the method of extracting and evaluating narratives “MAIN: Multilingual Assessment Instrument for Narratives” (Gagarina et al., 2019). This method was developed and validated in a Russian sample for children from 3 to 10 years old (Akhutina et al., 2024; Gagarina et al., 2019), and adapted for more than 20 languages. In addition, when assessing the macrostructure of the narrative, the criteria developed by Akhituna for semantic completeness and adequacy of speech, were also taken into account. These criteria are complementing the MAIN methodology (Akhutina et al., 2024).

Series of pictures equivalent in content were presented to the child: “Nest” on the pre-test and “Baby goats” on the post-test). Both series include a sequence of 6 pictures that combine into 3 episodes. To carry out the methodology, the series were printed out and folded into a “layout book”. The child was given to look at this book and was offered the following instruction: “Now I’ll show you the comics. Do you like comics? Look. What happened here? Tell me a story. Tell me as much as you can.” The child’s narrative was audio-recorded. After the transcription of the audio recordings, the total number of words in the child’s story was counted, the pace of speech as the ratio of the number of words to the time of the story, and the macro- and microstructure of the story (narrative) was evaluated.

The macrostructure of the story (maximum 10 points) includes 2 subscales rated from 1 to 10 points (the final score is calculated as the mean of two subscales): story programming (semantic completeness, internal consistency and adherence to the narrative structure “goal – action – outcome”) and semantic adequacy (the criteria developed by Akhituna: matching the story to the presented pictures, understanding cause-and-effect relationships). Microstructure (maximum 10 points) also includes 2 subscales rated from 1 to 10 points (the final score is calculated as the mean of two subscales): the lexical design of the story (literacy in the lexical use of words, morphological diversity) and the grammatical and syntactic design of the story (grammatical and syntactic errors, grammatical and syntactic diversity).

### Intervention

In the PlayWorld group, pretend play based on the plot of a fairy tale with the participation of an adult as a play partner was organised (Fleer, 2022). The participants were offered a short version of the fairy tales «Pinocchio» (1–11 play sessions) and “The Wizard of Oz” (12–22 play sessions). The experimenter read fairy tales to the children by chapter, then he asked some questions about the story (*e.g.* “Do you like the story?”, “Who is your favourite character?”, “Why did Pinocchio do that? How did he feel?”), and suggested playing the story. The children chose the roles, one role was assumed by the experimenter. Participants moved into the play world through the “portal”, selected suitable attributes for the role (fabrics and ribbons of different colors, hats etc.) and began to play. Children could use open play materials in the play space such as boxes, colored yarn, plastic tableware, chairs, pillows, coloured fabrics, sticks, colored cardboard, pebbles, etc. The experimenter maintained the plot and richness of the pretend play. At the same time, exact adherence to the plot of the book was not mandatory.

In the Free Play group*,* the adult helped children to start playing. He helped arrange the discussion of the topic, assign the roles, choose the plot and did not intervene anymore. The experimenter monitored the safety of children’s activities. The Free play took place in an environment enriched with open play materials same as in PlayWorld group. Open play materials were used to diversify the possibilities of implementing various themes and roles, as well as to encourage children to use substitute subjects in pretend play.

In the Control group no special play sessions were organised. The children attended kindergarten as usual.

### Data Analysis

All statistical analyses were conducted using Jamovi version 2.5.7. One-way analyses of variance (ANOVA) were conducted on the groups to test whether there were any differences between study groups and schools at pre-test; and on sex to control for possible sex-dependent differences in narrative development. The data were screened for normality and homogeneity of variances prior to the main analyses. The Shapiro– Wilk test was used to assess the normality of distribution, and Levene’s test was applied to evaluate the homogeneity of variances. These tests were used to determine whether the assumptions for parametric statistical methods were met. Parametric analyses (ANOVA and repeated-measures ANOVA) were conducted only when both assumptions were satisfied. In cases where these conditions were not met, non-parametric tests, such as the Kruskall–Wallis test, were used instead.

To assess the effectiveness of the intervention, repeated-measures ANOVA using data from the participants who completed the tests in both time points were applied. Intervention and time were used as the independent factor to explore the interaction effects. There were 3 levels of the intervention as factor: PlayWorld group, Free Play group, Control group. There were 2 levels of time as factor: pre-test and post-test. When significant main effects or interaction effects were identified, post hoc tests were conducted to specify the nature of the effects, including within-group comparisons (pre-test vs. post-test scores) and between-group comparisons at post-test. Bonferroni corrections were applied to control for the increased risk of false positives (type 1 errors) when performing multiple comparisons on the same dataset. Effect sizes were reported using partial eta squared (η^2^).

The significance was established at a p value of .05 throughout the analysis.

## Results

### Preliminary analysis and descriptive statistics

Multifactorial ANOVA did not reveal any significant differences between study groups at pre-test for any coherent speech indicators (ANOVA, p > .05 for each indicator). No significant differences were found between the participating kindergartens at the pretest stage for any of the measured indicators (ANOVA, p = .538 for word count; p = .24 for pace of speech; p = .763 for the macrostructure; p = .621 for the microstructure). This suggests that the baseline level of narrative competence was comparable across settings, allowing further analyses to proceed without controlling for institutional affiliation. Both at pre-test and post-test data on pace of speech, macrostructure, and the microstructure of the narratives in the groups is normally distributed and have equal variances. For the number of words in narratives the data is distributed abnormally (Shapiro-Wilk criterion, W = .833, p < .001). Non-parametric tests were used in further analyses for the number of words. *Table 2* provides an overview of descriptive statistics, including the mean scores, standard deviation for each indicator at pre- and post-test for each of the study groups, the results of the ANOVA for group differences, the results of the Shapiro-Wilk test, and Levene’s test. There were also no significant differences in the preliminary assessment between boys and girls (ANOVA, p > .05 for each indicator). No differences in gender composition were found between the groups (χ^2^(2) = 2.28, p = .32). Therefore, a further analysis of group differences was carried out without limitations.

**Table 2:** Descriptive Statistics for every coherent speech indicator before (pre-test) and after the intervention (post-test) in different study groups

Coherent speech indicators in each study group	Pre-test M±SD	Shapiro- Wilk test	Levene’s test	Post-test M±SD	Shapiro- Wilk test	Levene’s test
*Number of words in the narrative*						
PlayWorld group, N = 22 Free Play group, N = 25	48.8±13.5 48.5±25.2	W= .82, p< .001	F(3,55)= .285, p= .754	51.9±15 52.7±16.3	W= .964, p= .107	F(3,55)=2.29, p= .112
Control group, N=20 Kruskel-Wallis test, p	43.6±12.9 p = .61			50.3±21.9 p = .869		
*Pace of speech*						
PlayWorld group, N = 22 Free Play group, N = 25	.922± .292 .875± .281	W = .98, p = .45	F(3,55) = 1.32, p = .278	.959± .19 .92± .205	W = .968, p = .163	F(3,55) = .78, p = .925
Control group, N=20 ANOVA, p	.687± .173 p = .072			.912± .262 p = .777		
*Macrostructure of the narrative*						
PlayWorld group, N = 22 Free Play group, N = 25	5.32±1.2 4.65±1.17	W= .972, p= .193	F(3,55)= .857, p= .469	6.09±1.07 5.47± .9	W = .986, p = .8	F(3,55) = .04, p = .955
Control group, N=20 ANOVA, p	5.11±1.27 p= .563			3.79±1.47 p< .001		
*Microstructure of the narrative*						
PlayWorld group, N = 22 Free Play group, N = 25	4.95±1.27 4.35±1.17	W= .977, p= .318	F(3,55)=1.87, p= .145	6.25±1.14 5.58±1.1	W = .982, p = .596	F(3,55) = 3.16, p = .079
Control group, N=20 ANOVA, p	5.56±1.13 p= .563			4.57±1.2 p< .001		

### Analysis of the Intervention Effects on Children’s Narrative Competence

Repeated-measures ANOVA examined main and interaction effects of the intervention factor on the development of narrative competence indicators (pace of speech, macro- and microstructure of the narratives) and its change over time. The Kruskel-Wallis test was performed to evaluate the mean differences between the number of words in the narratives at pre- and post-test.

The main effect of time at the trend level was found for the pace of speech (F(1,42) = 3.85, p = .056, η^2^ = .034). No differences were found between study groups.

**Figure 1.**
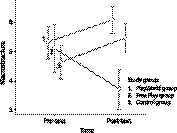
Group marginal means for the macrostructure of narratives at pre-test and post-test

For the macrostructure of the narratives, a significant interaction effect of time and intervention was depicted (F(2,51) = 8.049, p < .001, η^2^ = .141, *see Figure 1*). Post Hoc analyses revealed a significant increase from pre-test to post-test for the Play-World group (t(21) = –2.167, p_Bonferroni_ = .044) and Free Play group (t(24) = –2.421, p_Bonferroni_ = .028). Children in Control group showed a significant decrease in scores from pre-test to post-test (t = 3.239, p_Bonferroni_ = .032). Children from both the Play-World group (t = 5.89, p_Bonferroni_ < .001) and Free Play group (t = 3.76, p_Bonferroni_ = .006) showed higher scores at post-test than children in Control group. Children from the PlayWorld group at the trend level showed better scores at post-test than children from the Free Play group (t = 3.005, p_Bonferroni_ = .062).

**Figure 2.**
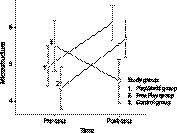
Group marginal means for the microstructure of narratives at pre-test and post-test

For the microstructure of the narratives, a significant interaction effect of time and intervention was revealed (F(2,51) = 8.808, p < .001, η^2^ = .148, *see Figure 2*). Post Hoc analyses showed that children from the PlayWorld group showed significantly higher scores at post-test than children in Control group (t = 5.89, p_Bonferroni_ < .001) and at the trend level higher than in Free Play group (t = 3.063, p_Bonferroni_ = .052). Children from the PlayWorld group (t(21) = –3.458, p_Bonferroni_ = .003) and the Free Play group (t(24) = –3.497, p_Bonferroni_ = .003) performed significantly better at post-test than at pre-test.

For the number of words, no significant differences were found between the groups in the gain scores from pre-test to post-test (Kruskel-Wallis test, χ^2^(2) = 2.03, p = .362).

## Discussion

This study aimed to assess the effectiveness of PlayWorld interventions in fostering preschoolers’ narrative competence. We compared the influence of free pretend play and experimental intervention consisting of PlayWorld which incorporated joint adult–child pretend play of a text from children’s literature and general discussion. We also compared this PlayWorld practice with a control non-intervention group. The results have shown that children both from the PlayWorld group and the Free Play group performed better at post-test than children from the Control group in terms of macrostructure of narratives. Moreover, children who did not attend play sessions showed significant decline of scores for the macrostructure of narratives. Results also indicated that children from the PlayWorld group performed better at post-test than the Control group and at the trend level better than in Free Play group. Comparing adult-guided pretend play based on cultural stories (PlayWorld framework) with free pretend play allows for a deeper understanding of what exactly creates the conditions that lead to the narrative development in play.

Both pretend play interventions revealed significant impact on the semantic level (macrostructure of the narrative) of narrative development in young children. The obtained data align with previous studies demonstrating the positive impact of play on speech development and further expand upon them (Griswold, 2007; Stagnitti & Lewis, 2015). Specifically, it appears that the nature of pretend play itself contributes to the development of the communicative function of speech and the advancement of its semantic level. Apparently, a fundamental reason for the narrative development in play is its intrinsic narrative nature, as it involves exploring the roles and constructing different realistic or fantasy scenarios (Hakkarainen & Bredikyte, 2014). Pretend play and storytelling, in this context, can be seen as interconnected forms of narrative expression, positioned along a spectrum that ranges from verbal storytelling to the physical enactment of narratives in play (Nicolopoulou, 2015). However, in pretend play, the physical enactment of a narrative occurs through the creation of an imaginary situation. The child is not merely fantasising or acting out a performance but simultaneously maintains both real and imaginary levels of action (Vygotsky, 2004, 2016). The term “imaginary” refers to the child’s attribution of new meanings to what is directly perceived — what children commonly describe as “make-believe” or “as if.” It is important to emphasise that the imaginary situation in play does not exist solely in the children imagination but is realised through their practical actions, either individually or collaboratively (Smirnova & Ryabkova, 2010). Pretend play is impossible without the maintaining both the imaginary and real levels of action. These two levels form the dialectical structure of pretend play (Veraksa, 2022). The dialectical structure of pretend play serves as an internal condition for the emergence of two levels of communication (Bluiett, 2009; Fein, 1979; Veraksa, 2022). Firstly, pretend play is structured around roles and imaginative scripts with dialogues. Children relate to one another in the roles they agreed to perform, exploring different aspects of the characters and their goals, plot and actions. Secondly, pretend play implies a collaborative nature of interaction about the play — children negotiate the roles, create the plot, discuss how to organise the game, and regulate play actions. Together these two aspects of play enhance the development and awareness of the communicative function of speech, highlighting the communicative goals.

Additionally, pretend play involves the appropriation of the semantic aspects of culture and social relationships (Veraksa et al., 2023). It serves as a shared space of meanings for its participants, where play actions are directed toward constructing relationships with cultural artifacts. A.N. Leont’ev, analysing the example of a child playing with a stick (Leontiev, 1997, pp. 479–480), demonstrated that the child’s play action reproduces the cultural action of an adult in terms of its goal. However, play actions are characterised by only partial alignment with real adult actions. This suggests that, due to the difficulties in mastering the operational aspects of adult actions, the child primarily internalises the semantic dimension of the action in play. Thus, pretend play contributes to the semantic development of the narratives.

The conditions created in a PlayWorld scenario, according to the findings, make a significant contribution to the development of the lexical-grammatical level of speech production (microstructure of the narratives). In contrast, the development of the lexico-grammatical level of speech production in the free pretend play did not differ significantly from natural development without intervention. Reenacting cultural text and participation of adult in pretend play as a genuine player, who maintains the plot without introducing additional educational practices, significantly enhance lexical-grammatical development in children. Pretend play and language are both of a symbolic nature (Bourdieu, 1991; Vygotsky, 2017; Veraksa, 2011). As highlighted by Vygotsky (Vygotsky, 2017) play serves as a space to imagine and enact roles within a constructed fantasy scenario, promoting greater maturity in children use of language and gestures. During pretend play, children use objects, language, and gestures not in their literal sense but as representations of absent objects, actions, or concepts. Likewise, language serves as a system of symbols that enables individuals to label objects, convey emotions, and articulate thoughts (Bourdieu, 1991).

The worlds of play — the “playworlds” are based on the cultural texts, which depicts common child experiences (Fleer, 2022; Hakkarainen & Bredikyte, 2020). Common experience, joint feelings, dramatic collisions in social relations between role characters stimulate shared play ideas and shared “perezhivanie” or “intensely-emotional-livedthrough-experience” (Ferholt, 2010), while in free pretend play children lack joint sense-making (Hakkarainen & Bredikyte, 2019; Veresov, 2017). Shared “perezhivanie” means that children are inside the problem situation, emotionally and cognitively involved. Thus, shared “perezhivanie” allows children to immerse deeper and longer into the role and the plot, fostering role communication and discussions about the process of play.

In addition, in the PlayWorld enactment of stories, adults bring their culturally accumulated experiences to the play activity (Lindqvist, 1995). The adults contribute interpretations and ways of the use of substitute objects and symbolic means, the intonation of characters, lexical diversity, including the use of the poetic language of the story as well as the use of aesthetic forms (scripts, props, stage effects, costumes, and so on). In particular, an adult can initiate dialogues between the characters more often than children in the play. Moreover, adult participation allows to hold an imaginary situation longer, while in a free pretend play, children quickly “fall out” of an imaginary situation, switching to impulsive and field actions. Thus, joint child-adult play and cultural story background created conditions for the development of the lexical-grammatical level of speech.

Although child’s play is always considered his/her sacred space and adults are not allowed to step into it, results indicate the crucial role of adult involvement in pretend play who helps maintain children’s engagement in the imaginary situation. Prolonged and complex play provides valuable opportunities for cultural knowledge acquisition and narrative development (Andresen, 2005; Elkonin, 2005). However, according to the cultural-historical approach, any children activities, as well as play, may not achieve a high level of development without adult guidance (Veresov et al., 2021; Samuelsson, 2020). Rich and well-organised play requires diverse skills from children such as planning and organisational skills, creating engaging narratives, choosing the appropriate attributes, organising the game space, solving problems between players, maintaining dramatic colossi, and developing the plot. Since these abilities lie within the child’s Zone of Proximal Development (ZPD), adult guidance is essential for their progression. This suggests that adult participation may create structured conditions for practicing and strengthening language, thereby increasing the developmental potential of play.

An unexpected decrease in narrative macrostructure scores was observed in the Control Group at post-test. Several possible explanations may account for this finding. First, in the absence of targetted language stimulation, preschoolers may lack sufficient opportunities to practice coherent, structured speech within the typical educational environment. Minor regressions in skill development are not uncommon in early childhood and may reflect natural fluctuations in developmental trajectories, particularly when supportive interventions are absent (Vygotsky, 1978). Second, periods of cognitive overload, stress, or changes in daily routines— common in preschool settings — can temporarily disrupt children’s speech production and reduce their ability to construct coherent narratives. Thus, such changes may be attributed to random effects.

## Conclusion

This study presents an attempt to examine the effectiveness of PlayWorld framework and analyse conditions of pretend play that foster narrative development in children. The findings indicate that pretend play contributes to the development of the semantic level of speech production. The developmental effect of pretend play appears to be associated with its dialectical structure, which requires the child to operate on two levels— real and imaginary. Furthermore, the study demonstrates that joint child-adult pretend play based on a fairytale within the PlayWorld framework, also supports the development of lexical and grammatical aspects of speech through the shared emotional “perezhivanie”. In addition, the adult helps the child to hold both real and imaginary levels of play for a longer period, thereby reinforcing the symbolic and communicative functions of speech; and introduces cultural experience into the play, demonstrating various ways of using language within the play context. This study opens new avenues for further research on the internal processes of pretend play for child development. The findings can be implemented in early childhood education settings to create conditions that foster the development of coherent speech in preschool children.

## Limitations

The primary limitation of this study concerns the reduction in sample size from the pre-test to the post-test, which could potentially diminish the reliability of the statistical conclusions. To address this factor, the final sample groups were controlled for equivalence in the assessed parameters at the initial diagnostic. However, accounting for additional control variables could have identified other factors influencing speech development and yielded more robust results. Finally, a limitation of the present study is that the reported results reflect only the immediate effects following the intervention. No delayed post-intervention assessments were conducted, which limits the ability to determine whether the observed improvements in narrative competence were maintained over time.

Future research should incorporate follow-up evaluations to examine the long-term sustainability of the PlayWorld effects on narrative competence. Furthermore, designing experimental studies that more precisely isolate the underlying developmental mechanisms would help clarify their specific contributions and improve the effectiveness of intervention programs. Additionally, including control variables such as verbal fluency, cognitive processes, or bilingualism could provide a more nuanced understanding of factors influencing development
